# A decade of Does: celebrating the 125th anniversary of MLA through an annual meeting conversation with past Janet Doe lecturers

**DOI:** 10.5195/jmla.2025.2150

**Published:** 2025-04-18

**Authors:** Gerald Perry, Mary Joan (M.J.) Tooey

**Affiliations:** 1 jerryperry@arizona.edu, Associate Dean Emeritus, University Libraries, University of Arizona, AZ; 2 mjtooey@hshsl.umaryland.edu, Library Faculty Emerita, Health Sciences and Human Services Library, University of Maryland, Baltimore, MD

**Keywords:** Medical Library Association, History, Janet Doe Lecture, Ethics, Professional, Libraries, Leadership, Diversity, Equity, Inclusion, Health Equity, Technology, Open Access Publishing

## Abstract

At the Medical Library Association (MLA) 2024 Annual Meeting in Portland, Oregon, the Janet Doe Lectureship Series plenary session featured a panel of past Doe lecturers from the last decade. Reflecting on their lectures they were challenged to imagine how the Association's Core Values could guide and inform decision making in response to current and emerging challenges to the profession and in the environment. Panelists' reflections included themes of inclusivity, collaboration, leadership, technology, space planning, and the role of medical librarians in addressing issues of mis- and disinformation, bias, equity, and open access, today and in the future. Common themes included the centrality of collaboration as a necessary component of health sciences librarianship, and the ongoing criticality of the profession's commitment to ethical practices. The panelists shared insights on how MLA's Core Values can guide the profession and association through the challenges and opportunities of the evolving healthcare and information landscape, including the rise and the rapid evolution of advanced technologies.

## INTRODUCTION

The Janet Doe Lectureship series was established in 1966 to honor Janet Doe, former librarian of the New York Academy of Medicine and past Medical Library Association (MLA) president. The series aims to recognize individuals for their unique perspectives on the history or philosophy of medical librarianship. The Lectureship is a standing plenary session as part of the MLA Annual Meeting, featuring a single committee-vetted speaker nominated by association membership. Since 2022, the Annual Meeting has been a hybrid in-person and online meeting.

For 2024, circumstances necessitated a change in the standard single speaker format and instead featured panel presentations from lecturers from the last 10 years. The session was moderated by a library director colleague, J. Dale Prince, who introduced the panelists and led the question-and-answer period following the panelists' initial comments.

This article describes and summarizes the perspectives shared by the panelists. Given as a prompt before the session, panelists were asked to reflect on their lectures and address how MLA Core Values could guide and inform decision-making in response to current and emerging challenges to the profession and in the environment. Panelists were given the option to pre-record a video of their comments; one did. Panelist comments were limited to five minutes or less. The session was organized as a conversation, and panelists responded to questions from the in-person and virtual audience.

## PANELISTS

J. Dale Prince served as moderator. Panelists included:

- Margaret Bandy, AHIP, FMLA (Doe Lecturer 2014 https://pmc.ncbi.nlm.nih.gov/articles/PMC4279930/);
- Barbara Epstein, AHIP, FMLA (2015 https://pmc.ncbi.nlm.nih.gov/articles/PMC4722639/);
- MJ Tooey, AHIP, FMLA (2016 https://pmc.ncbi.nlm.nih.gov/articles/PMC5234446/);
- Elaine Russo Martin, FMLA (2018 https://pmc.ncbi.nlm.nih.gov/articles/PMC6579597/) represented by Tooey;
- Gerald Perry, AHIP, FMLA (2019 https://pmc.ncbi.nlm.nih.gov/articles/PMC6920003/);
- Chris Shaffer, AHIP, FMLA (2020 https://pmc.ncbi.nlm.nih.gov/articles/PMC7772973/);
- Sandra Franklin, AHIP, FMLA (2021 https://vimeo.com/1035668889/f00e05408cshare=copy);
- Michael R. Kronenfeld, AHIP, FMLA (2022 https://pmc.ncbi.nlm.nih.gov/articles/PMC10259618/); and
- Michelle Kraft, AHIP, FMLA (2023 https://pmc.ncbi.nlm.nih.gov/articles/PMC11189140/).

The panelists spoke in chronological order based on the date of the receipt of their award of the Doe Lectureship.

## PANELIST PRESENTATIONS

Margaret Bandy focused on pivoting for a more inclusive and collaborative MLA. She discussed the concept of pivoting encouraging hospital librarians to confront challenges and find new ways to thrive in their organizations. She referenced a meditation by Deng Ming-Dao [https://dengmingdao.com], emphasizing the need to change one's life and form new paths. She shared her experience of collaborating with healthcare professionals and moving outside library walls, inspired by MLA Past Presidents Elaine Russo Martin and Jane Blumenthal.

Leadership qualities informing the past, present, and future of MLA were addressed by Barbara Epstein. For her 2015 lecture, oral histories of MLA presidents were reviewed to identify recurring themes and lessons for contemporary practitioners. Generational shifts in the profession and the importance of collaboration and group work in achieving organizational goals were noted. She shared her experience on a task force identifying MLA notables and the realization that in recent years the work of the association is mostly advanced by groups rather than individuals. The foundational role of MLA values in guiding professional leadership and decision-making were emphasized by M.J. Tooey within the context of the qualities of past and contemporary MLA leaders. Tooey highlighted the need for medical and health sciences library leaders in embracing and modeling MLA values, especially in the context of artificial intelligence (AI) and related advanced technologies. She challenged attendees to boldly embrace and live MLA values in their leadership roles, emphasizing the importance of integrity and collaboration.

Elaine Russo Martin was unable to attend the meeting but shared her presentation with Tooey before the session. Tooey spoke on Martin's behalf. Martin addressed the concept of spatial justice and its importance in library renovation projects. She shared her experience leading a multi-year, multi-million-dollar renovation of the Harvard University Countway Library of Medicine [https://countway.harvard.edu/renovation], incorporating social justice ideals into the newly renovated space. The importance of community dialog, environmentally friendly building materials, biophilia, accessible spaces, inclusive seating, and engaging diverse voices in library design were stressed. She underscored the role of library spaces in promoting inclusivity, accessibility, and community engagement.

The importance of ethics and health equity in advanced health information technologies were discussed by Gerald (Jerry) Perry. He focused on the need for librarians to address potential biases in AI and other advanced technologies and to advocate for equity in their application. Perry shared his involvement in the Mobilizing Computable Biomedical Knowledge (MCBK) movement [https://mobilizecbk.med.umich.edu] and the importance of centering equity in health information technology. He encouraged attendees to engage in MCBK-related work and to consider the ethical implications of AI and other advanced technologies in healthcare.

Chris Shaffer discussed the long history of medical librarians in promoting barrier-free open access to information and the importance of sharing knowledge and data. He highlighted the role of medical librarians in developing technologies like Web of Science, Medline, and services such as interlibrary loan. Shaffer emphasized the need for open science and data sharing to ensure that health information is accessible and transparent. He expressed concerns about the potential negative impacts of AI on open access and the importance of librarian involvement in AI projects to ensure equitable and informed use of data.

The importance of diversity, equity, and inclusion in MLA and the broader health sciences community were stressed by Sandra Franklin. She highlighted the establishment of special interest groups (SIGs) and the importance of caucuses in MLA in support of diverse communities and in fostering a sense of community within the Association. Franklin emphasized the need for MLA to continue to value and support accessibility for all stakeholder groups. She shared her experience as chair of the MLA Diversity and Inclusion Task Force and the impact of their work on MLA's values and initiatives.

While unable to attend the meeting, Mike Kronenfeld shared a pre-recorded video wherein he discussed the transition from print-based to digitally based health information environments and the role of health information professionals (HIP) in this transition. He emphasized the importance of upgrading skills and services to support the emerging data-driven health information ecosystem. Kronenfeld highlighted the need for HIPs to understand the significance of the transition and to continue developing their skills and knowledge. He encouraged MLA to support the profession's transition by continuing to develop overviews of the digital health information environment and the role of health information professionals.

Michelle Kraft focused on the challenges of educating others to find quality health information in the face of fake news and the importance of AI in this context. She emphasized the need for librarians to be aware of the accuracy and relevance of information and to help patrons use information for good. Kraft underscored the importance of understanding the data within AI and other resources and the potential for misuse of information. She encouraged attendees to be proactive in addressing misinformation and disinformation, continuing to explore ways to provide accurate and relevant information to their communities.

## DISCUSSION: PIVOTING, AN EVOLVING ASSOCIATION, AND THE CENTRALITY OF COLLABORATION AND A COMMITMENT TO ETHICS

Explicitly or implicitly, each of the panelists related the nature and impact of their work to the various collaborations and interprofessional engagements they experienced in their professional lives. They described an association that has grown and evolved over time, consistently in the direction of envisioning expanding horizons for the scope and nature of health sciences librarianship. They related a “growth mindset” attitude to the scope, nature, organization, and values of the profession. They also linked their work to the ethics of librarianship.

### Collaboration and Change!

Margaret Bandy, whose career was deeply rooted in hospital librarianship, encouraged her hospital librarian colleagues to collaborate with the healthcare professionals in their organizations. According to Bandy, “My experience was that the more I involved myself in their work, the more I and they learned how library services could support them. For me, my professional life was enriched by collaboration and professional organizations, and in my position at St. Joseph Hospital [Denver], sometimes I had to invite myself to the table in order to be included. Fortunately, all our meeting rooms had lots of chairs.”

Bandy relayed her experiences working with healthcare professionals outside of the library as a means of having greater influence and impact. Epstein also stressed the ability to collaborate and complete group work as a key leadership quality. She linked these abilities to the need for openness to change and adaptability. As Epstein noted in her Doe Lecture and in our panel, “Our profession is changing rapidly, and so is our association. We're seeing a generational shift as baby boomers move toward retirement, and a new cohort of medical librarians enter positions of leadership in the association and in our institutions. As this new generation creates the future, it's useful to remember that progress is rarely a smooth progression of preordained successes, but rather a series of false starts, wrong turns, frustrations, insights, ‘aha moments’ and course corrections.”

Epstein's comments aligned with Bandy's focus on the necessity of pivoting. Bandy stated, “As an organization, MLA has pivoted many times, often urged by members, as in the establishment of the Research Training Institute, but also by outside events. When COVID hit, MLA pivoted to online, virtual meetings and hybrid meetings. MLA pivoted by approving the motion by the Diversity and Inclusion Task Force requesting the addition of a new association value – of an open, inclusive, and collaborative environment within and outside the profession.”

Franklin's comments brought the audience deeper into the pivotal time and place in the life of MLA when diversity, equity, and inclusion finally took center stage.

I was honored to give the 54th Janet Doe lecture during the second virtual MLA conference in 2021. It was quite a time to be the lecturer - the deaths of Breonna Taylor, George Floyd and Dante Wright fresh in the news. Foremost, we were all impacted by the COVID 19 pandemic. We lived through a change in lifestyle under quarantine coupled with the loss of colleagues, friends, neighbors, loved ones and acquaintances. News headlines showcased unacceptable health disparities, conditions such as diabetes, hypertension and asthma that tend to plague African American communities more than other groups, adding to the COVID 19 death count. Income inequities and disparities in access to health care tend to hurt minority and lower income populations more than others, and that is still the case today. This was the environment in which we found ourselves in as medical and health sciences library professionals attending a virtual conference, supporting the transformation of our health systems and hospitals, medical and health sciences schools, and our society, while we too, examined diversity in our profession.

Franklin's reflections on diversity, equity, and inclusion as values of the profession were rooted in the ethics of the association. Franklin drew a direct connection between the emergence of the new DEI value and the emergence and growth of identity-based caucuses within the Association. She noted,

The African American Medical Librarians Alliance (AAMLA) had its beginning at a dinner in New Orleans during the 1988 annual meeting. AAMLA officially became a SIG in 2000 at the Vancouver, Canada annual meeting, and any of the AAMLA members can tell you about all the wonderful initiatives that AAMLA sponsors on a regular basis every calendar year. The Hispanic SIG held its first meeting in May 2014, and our MLA President Elect candidate, Brenda Linares, was among the founders of what is now the Latinx caucus. A caucus for the Asian American and Pacific Islander communities was proposed at the time of my lecture in response to acts of violence against the Asian community. That caucus is now joined with the social justice and health disparities caucus. These caucuses provide members with community within MLA. Caucus participation allows MLA members to assume leadership roles, lead initiatives, and make our MLA memberships richer for the colleague connections they create.

### Moral Leadership

Tooey's work to understand the common traits of leadership surfaced the centrality of professional ethics as essential to advancing mission-based goals. She told the audience,

The [Doe] lecture became a springboard for me for learning, exploring, and teaching, and one of the things I learned was that truly good, effective, and impactful leaders have strong moral beliefs and values and foundations, and that is where MLAs values come in for all of us. They are foundational to the way we do our work and the way we lead within our careers, our institutions, our professions, our professional home, MLA and even the way we live. They are our moral compass, and they tell us what we stand for. MLA values evidence. We value high quality information, lifelong learning, research and evidence-based practice, inclusion and collaboration, and accessibility for all. We have ethical standards. We even have a code of ethics stating that knowledge is essential for informed decision making in healthcare, education, and research, whether it is for society, clients, institutions, our professions, or ourselves. And diversity, equity and inclusion are values foundational and supporting absolutely everything.

Martin's 2018 Doe lecture was grounded in the ethics of the profession and the moral argument for social justice. Her comments called for equity. She updated her original thoughts by relating her efforts around equity through facilities planning, work that was deeply grounded in centering the interests of clients. Martin asked, “How can we create library spaces promoting our democratic ideals, equity, accessibility, community, engagement and the health and welfare of our users and social justice Understanding the relationship between library space and social justice is imperative as many of us embark on renovation projects in our libraries.”

According to Martin, “Since COVID, library spaces have become even more important as gathering spaces and social hubs for our campus community. With remote work, hybrid work and return to work initiatives, our users are yearning for a place to go and reconnect with their friends and colleagues. The library can serve this need.” Describing her building renovation project, Martin said, “We embraced user design and user participation principles in the planning process and the conceptual redesign of spaces. We co-created spaces. We found ways to gather feedback and engage the community in dialog about library spaces through focus groups, feedback sessions and furniture fairs… We committed to only using healthy building materials and worked with vendors to adopt these into their products. We incorporated biophilia, ways to connect with nature, into the design. We used sustainable building materials such as terrazzo flooring instead of carpeting and incorporated natural light and wood into building design.”

Perry's 2019 lecture picked up on Martin's social justice theme and conveyed its impacts on his personal and professional life. For the panel, he related his most recent efforts advancing librarian engagement in computable biomedical knowledge, noting that this work was a continuation of his social justice work, similarly predicated on a commitment to the ethics of care. He noted,

In recognition of the 125th anniversary of our association and this particular event, I wanted to talk about the value of ethics and center my comments on that particular and specific value. The core of my Doe lecture was on the concept of equity. And at the time, I had been thinking about equity for LGBTQ+ plus people. I had also been thinking about the history in the association around equity for librarians. But lately, I've been thinking about this concept of equity in advanced technologies and the work of mobilizing computable biomedical knowledge. When we think about advanced technologies and equity, we need to think about making sure that these technologies correct for the potential for bias to exist and address the potential negative impacts of that bias especially on communities who are traditionally marginalized and minoritized. I believe we have a key role to play in that space. When we think about the work that librarians do, when we work with metadata, when we work with preservation, when we look at things like the FAIR Principles [https://go-fair.org/fair-principles] and CARE Principles [https://gida-global.org/care], finding information and getting information to the right hands at the right moment, at the right time, all of this work, really is work that we're familiar with as librarians. But we haven't seen librarians come to the MCBK table to date, very robustly.

Shaffer reminded us of the centrality of open access as an ethical stance hinging on effective partnerships across the information ecosystem. According to Shaffer, “In 2020 I spoke about the long history of the Medical Library Association and medical librarians in bringing information to the communities they serve, whether those are health professionals, patients, caregivers, in public health environments and so forth. We have been moving towards open and pushing towards open access to information, to free and accessible information, since the very beginning of our association.” He went on to ask, “Why are we giving the results of health sciences research, of information about the best way to care for people when they are at their most vulnerable, to commercial entities to exploit and to sell back to us ” Shaffer suggested, “As we think about the principles of open science and open access, we also need to consider the principle that information about how to take care of people, how to help patients, how to make people be healthier, how to encourage people to live healthier lives, should not be locked up. And the movement of open science is a continuation of that, the idea that all of the information that we're using in order to do this research should be shared, from the very beginning of an experiment through the end, that there shouldn't be hidden information.”

### Values that Guide Across Time

Reflecting on how the values of the Association and specifically the value of practicing our profession in an ethical manner, Tooey said, “We need our values to guide and underpin our professional leadership decisions. Small and large, we need to be models of integrity, leading through example in all our leadership roles. We can't afford not to do so. After all, we have an entire Association behind us. I challenge all of us to boldly embrace, model, and live those values compassionately, courageously, and completely as we embrace all the leadership roles we undertake.”

According to Epstein, “As I reviewed MLAs core principles and values, I have to conclude that though the goals have evolved through the years, the themes remain constant: promotion of scientific evidence and access to high quality health information along with reliance on evidence-based practice ethical principles and lifelong learning.”

Kronenfeld's comments also made note of changes but also consistency – for the Association and the profession – across the span of time. He reminded us of the many critical historical collaborations and partnerships with entities such as the US National Library of Medicine that have informed the work of the association across the decades. According to Kronenfeld, “With the emergence of the digital information environment, we are faced with a challenge and the opportunity in our transformation from medical librarians facilitating access and use of the print based, knowledge-based information collections provided by our libraries to new roles as health information professionals (HIP). We are now HIPs working collaboratively with the units and staff we support, facilitating the effective access, use and management of digitally based, increasingly accessible, KBI/data.”

Kronenfeld acknowledged the leadership role of MLA when he noted, “Led by their professional organizations, the Medical Library Association and the Association of Academic Health Sciences Libraries, medical librarians, HIPs, need to understand the significance of the transition from the print to the digital health information environment, the shift of medical librarians to HIPs, who have been becoming more closely embedded in the programs and research teams they work with over the last 20 years. This has represented the start, and the shift from narrow support staff to that of full collaborators, as the data driven health information ecosystem being guided by NLM continues to emerge. It is crucial that the skills and services of our HIPs continue to upgrade and develop.”

As the last panelist but most recent Doe lecturer, Kraft reminded us of the “forever” ethics-based work of remaining vigilant to the harmful impacts of dis- and misinformation in the larger health information ecology. Kraft said, “I would like to challenge everybody…to look at how the information we are providing or the information that our patrons are using is accurate and relevant, and how that information can be used to influence healthcare, healthcare policy and other policies. We need to be aware of how it can be misused, not only as misinformation and disinformation, but through the twisting of information, perhaps very reliable information, to further an agenda. These are things that we as librarians, as information specialists have the ability to help people with - to use the power of information for good, to understand how it can be used and how it can be changed.”

Shaffer echoed Kraft's comments, noting, “What do we do when [bad actors] inject misinformation at a real deep scale for science into the systems that we're using that we don't understand how they work. We don't know exactly what's going to come out of them… I challenge all of you, especially the younger IT-oriented librarians in the room to start looking for ways to influence this.” Shaffer also spoke to the challenges brought about by the advent of AI at scale, for instance through large language processing systems. “At every institution that we work at, somebody is doing something with AI and librarians should be there, arm in arm with them, figuring out how to make these things equitable, figuring out how to make them informative and figuring out how to keep open open.”

Tooey further challenged the audience to be vigilant to the challenges ahead: “Unless medical or health sciences library leaders embrace and model the solid values and foundations espoused by MLA, the leadership attributes I have mentioned ring hollow and are, a) hard to achieve; b) potentially worthless; and c) could spell the end of our profession in these days of misinformation, disinformation and downright lies.”

The session concluded with audience questions and panelist responses.

## DISCUSSION: AUDIENCE QUESTIONS

J. Dale Prince as program moderator fielded questions from the audience and relayed those received via the online meeting platform to the panelists.

*What might you have done differently in your career*

Shaffer started the discussion by saying, “The main thing I would have done is to be more aware of the human relationships with the people I was working with, taking more time to get to know people as people, especially patrons.” Franklin said that she wished she had more deeply engaged in research. “Now with data science and AI, it's really important that librarians have research skills and get out there and be equals, if you will, in that level of the community, so that we can talk the talk and show examples of doing the work to have credibility within our spaces.” Perry said he wished he had pursued an advanced degree in natural language processing, a long-held interest that is related to his current engagement with computable biomedical knowledge.

Kraft took a philosophical approach to the question and noted that she wishes she had learned not to self-limit but to strive for whatever she was interested in, or to not worry about limitations or boundaries. She said it was also important to have the grace and self-awareness that one can't do everything, to not be overly critical of ourselves, and to know we're good at what we do. She called out the need to celebrate our achievements and victories.

Tooey followed up on that notion, saying, “Celebrations are so important, acknowledgement of good and little victories, and not to obsess over things that, in the big scheme of things, 36 years later, did they matter No. Nobody died, you know, over anything that was a small mistake that I made… So being kinder and gentler on my successes, failures, and other people, listening to them, and being humble and kind.”

*What are your thoughts on providing leadership and direction in arenas that you may not fully understand or are changing so rapidly it's difficult to ever feel like you have a grasp of things*

Shaffer encouraged connecting with subject experts. He said, “One of the first things you have to do is find subject matter experts in that area that you trust. And they may be the people that are working for you or working with you, they may be other people, but you have to find a trustworthy source that can help you vet ideas and proposals and projects and so forth. And, of course, educate yourself. If I'm not in a position to understand a question well enough to answer it, is there somebody on my staff or somebody that I work with, who does, who I trust, and I can then go to them and say, What should we do, or talk to me about how we should make this decision.”

Franklin reflected on Shaffer's comments, noting that she took the risk of hiring subject experts who did not have a terminal degree in librarianship. She was convinced that having subject experts on her team would lead to greater impact and success. She noted that despite the criticism she faced she did not regret the decision.

Epstein said, “I think it's important for “leading in the dark” to have people that will tell you when you're wrong and tell you when you're going in the wrong direction, and won't be afraid to tell you the truth and tell you what's going on, as opposed to what you think you see is going on.”

*What are some suggestions for breaking the cycle of perpetual discussions of challenges in the profession*

Epstein reframed the question, stating, “I would say that we're not, when we talk about change, going around in the same circle. We're going around in circles that move forward.” Kraft added that this is the nature of work, stating, “There's always going to be challenges. If we don't have challenges, then I think there's something going wrong, because that means we're not evolving. That means we're not moving forward. We can't stay stagnant. So, to break the cycle of challenges, or change the cycle of challenges, I think we just need to do things one step at a time and keep moving forward.”

Perry appreciated the question and recognized the sense that as a profession we seem to talk about the same challenges over and over. He framed the issue as one for attention by library leaders, challenging them to talk to people who are expressing an interest in the profession, and understand what's their thinking and what is their approach He said, “There's all sorts of different ways that folks are coming forward now with expectations and understanding of the nature of their work and the kinds of things that they're looking to achieve. New people are questioning received notions about what it means to deal with hierarchies and work cultures. What it means to work with bosses who are functional or dysfunctional. To work in organizations that are healthy and not healthy, and to really look at that with eyes wide open.” He encouraged reading outside of the literature of the professional for fresh ideas to longstanding concerns.

*As the National Library of Medicine searches for a new leadership and a new vision, what characteristics and skill sets would you like to see in our new NLM director*

Tooey acknowledged that she is serving on the search committee for the leadership role, and said, “The new director of NIH Dr. Monica Bertagnolli has quite a vision and excitement about NLM. And making NLM the nexus of not only what used to be the publication literature, but also data, objects at NIH… I don't know what characteristics you'd want to see, but I know that there is great support at the uppermost levels of NIH.”

Kraft said, “I would like to see somebody who can encourage more collaboration between different types of libraries, including the hospital libraries.”

## CONCLUSION

Originally intended as a “one-off” event in recognition of MLA's 125th anniversary, audience feedback for the Decade of Does panel was very positive. It was even suggested that a similar, every decade session be held to stop and reflect collectively as an association. Overall, the session was the second highest rated plenary of the Portland annual meeting, according to attendee evaluations. Among the anecdotal comments found in attendee “my favorite session” meeting assessments was the statement that it was “so cool to have a bit of history and then application for the future.” One attendee noted that they “enjoyed having a panel and the excellent preparation and thoughtfulness of the presenters.” Another complimented the panelists as “down to earth.”

This feedback will be considered by future annual meeting planners. The panelists all agreed that it was an honor to participate and reflect on their original lectures while considering how MLA's values have and will continue to be a source of support and inspiration to our membership.

